# Process quality of decision-making in multidisciplinary cancer team meetings: a structured observational study

**DOI:** 10.1186/s12885-017-3768-5

**Published:** 2017-11-17

**Authors:** Pola Hahlweg, Sarah Didi, Levente Kriston, Martin Härter, Yvonne Nestoriuc, Isabelle Scholl

**Affiliations:** 1 Department of Medical Psychology, University Medical Center Hamburg-Eppendorf, Martinistr. 52, 20246 Hamburg, Germany; 2 Department of Psychosomatic Medicine and Psychotherapy, University Medical Center Hamburg-Eppendorf, Martinistr. 52, 20246 Hamburg, Germany; 3 Schön Klinik Hamburg Eilbek, Hamburg, Germany

**Keywords:** Cancer, Oncology, Multidisciplinary communication, Multidisciplinary team meeting, Tumor board, Decision making, Observation

## Abstract

**Background:**

The quality of decision-making in multidisciplinary team meetings (MDTMs) depends on the quality of information presented and the quality of team processes. Few studies have examined these factors using a standardized approach. The aim of this study was to objectively document the processes involved in decision-making in MDTMs, document the outcomes in terms of whether a treatment recommendation was given (none vs. singular vs. multiple), and to identify factors related to type of treatment recommendation.

**Methods:**

An adaptation of the observer rating scale Multidisciplinary Tumor Board Metric for the Observation of Decision-Making (MDT-MODe) was used to assess the quality of the presented information and team processes in MDTMs. Data was analyzed using descriptive statistics and mixed logistic regression analysis.

**Results:**

*N* = 249 cases were observed in *N* = 29 MDTMs. While cancer-specific medical information was judged to be of high quality, psychosocial information and information regarding patient views were considered to be of low quality. In 25% of the cases no, in 64% one, and in 10% more than one treatment recommendations were given (1% missing data). Giving no treatment recommendation was associated with duration of case discussion, duration of the MDTM session, quality of case history, quality of radiological information, and specialization of the MDTM. Higher levels of medical and treatment uncertainty during discussions were found to be associated with a higher probability for more than one treatment recommendation.

**Conclusions:**

The quality of different aspects of information was observed to differ greatly. In general, we did not find MDTMs to be in line with the principles of patient-centered care. Recommendation outcome varied substantially between different specializations of MDTMs. The quality of certain information was associated with the recommendation outcome. Uncertainty during discussions was related to more than one recommendation being considered. Time constraints were found to play an important role. Some of those aspects seem modifiable, which offers possibilities for the reorganization of MDTMs.

**Electronic supplementary material:**

The online version of this article (10.1186/s12885-017-3768-5) contains supplementary material, which is available to authorized users.

## Background

At present, multidisciplinary team meetings (MDTMs, also called tumor boards) are considered best practice in management and decision-making for cancer patients worldwide [1]. The National Cancer Institute in the United States defines a “tumor board review” as “a treatment planning approach in which a number of doctors who are experts in different specialties (disciplines) review and discuss the medical condition and treatment options of a patient” [2]. MDTMs are commonly organized by tumor entity, may vary in their team structure and typically consist of surgeons, oncologists, radiologists, pathologists, and in some cases, other health care professionals (e.g. specialist nurses) [1].

MDTMs evolved as a means to the end of good medical decision-making. The European Partnership Action Against Cancer consensus group defines MDTMs as an alliance of professionals “guided by their willingness to agree on evidence-based clinical decisions” [3]. However, malfunctioning MDTMs might lead to no recommendation being arrived at or documented [4].

Evidence on the effects of MDTMs on decision-making and clinical outcomes is mixed. The most proximal outcome of an MDTM is treatment recommendations. In a systematic review, Prades and colleagues found evidence that the implementation of MDTMs was associated with improvements in diagnostic and treatment recommendations for cancer patients with a variety of tumor entities [5]. It has also been found that MDTMs foster adherence to clinical practice guidelines (CPGs) [6]. As for more distal outcomes, a limited number of studies indicates that MDTMs improve clinical and process outcomes, for some tumor entities even survival rates, patients’ quality of life, their admission into clinical trials and the coordination of services [3, 5]. However, a large scale study from the US found little association of the existence of MDTMs with measures of use, quality, or survival, and therefore questioned the usefulness of MDTMs [6]. Designs and aims of studies on MDTMs have been greatly heterogeneous [7].

Bearing in mind that MDTMs are designed to positively impact clinical decision-making and patient management, it is crucial to explore the factors determining these processes more precisely [7]. This can be done by taking a closer look at the processes within the MDTM sessions. Lamb et al. outlined that the quality of information presented and the quality of teamwork are the two key components that are responsible for well-functioning MDTMs [8].

The information presented in a MDTM should cover medical information based on adequate radiological images and pathology results [8]. In light of a patient-centered approach, information on comorbidities, psychosocial aspects and patients’ treatment preferences should receive equal consideration when making treatment recommendations [3, 9]. The results of an observational study conducted by our team [10] and further publications [11, 12] suggest that information on comorbidities and the patient perspective is frequently not considered. Not taking into account these areas of information may result in recommendations that do not match the individual patient’s preferences or treatment recommendations that are not implemented [13].

In line with these findings, the treatment recommendation process in MDTMs was found to be mainly impeded by physicians having insufficient knowledge about the patient [11]. This includes for example the patient’s family status, his or her treatment preferences, and psychological distress. Some argue that patients themselves should be present at the MDTM discussion, but most physicians object to this [12]. If patients are not present themselves, this knowledge has to be brought to the MDTM by someone else, and needs to be acquired through sufficient patient contact before the MDTM. One suggestion to ensure this, is to have patient advocates at MDTMs (e.g. nursing staff), and that their input of the patients perspective at an MDTM should be heard in addition to the medical information discussed [13].

Additionally, it has been found that the composition of the participants and proper team work during the MDTM discussions are associated with effective MDTM functioning [9, 14]. The quality of team processes in MDTMs depends on interpersonal and behavioral skills of the participants, including a climate of respect between team members, good communication and an inclusive discussion [15]. Especially the chair of the MDTM holds a crucial role in promoting an open and communicative structure within the MDTM [14].

In summary, the quality of MDTM decision-making processes is highly variable [8]. Among other factors, it depends on the quality of the information presented and the ability to work together as a team. However, research regarding these factors is sparse [15] and has not yet been conducted in Germany. Only a small number of studies have assessed the quality of the presented information and team processes in MDTMs [8, 10, 12, 15]. Given the limited number of assessments of the quality of decision-making in MDTMs with standardized measures, the employment of a standardized measurement tool in MDTMs is required.

Thus, the aim of this study was to systematically assess the quality of decision-making processes at MDTMs. This included the following research questions: 1) Which type of information was presented and how was the quality of this information? 2) How was the quality of the team processes? 3) Which factors influence whether a recommendation is given at those meetings or not and whether one or more recommendations are given?

## Methods

### Study design

A cross-sectional, observational study was conducted. The study used a systematic observational assessment tool and a quantitative, explorative approach.

### Setting and subjects

Observations were carried out at different tumor-specific MDTMs at one University Cancer Center. The University Cancer Center Hamburg (UCCH) hosts 16 different MDTMs, most of them weekly, some every second or fourth week. Based on the results of a previous study [10], five MDTMs were excluded from this study, leaving an eligible sample of eleven MDTMs in the study. Criteria for exclusion were if the MDTMs merely discussed the status of patients rather than planned treatment, or had very few participants. Observations were conducted within the following MDTMs: dermatological, gastrointestinal, gynecological, head and neck cancer, liver and biliary tract, lymphoma and myeloma, neuro-oncological, non-entity-specific oncological, non-entity-specific surgical, thorax, and uro-oncological. All MDTMs were planned to be visited at least twice by one researcher (SD).

### Measures

An adaptation of the recently developed and validated observer rating scale Multidisciplinary Tumor Board Metric for the Observation of Decision-Making (MDT-MODe) was used for data collection. The measure has been developed by Lamb and colleagues [16] and has been well validated to assess the quality of the clinical treatment recommendation process in MDTMs [17]. The MDT-MODe assesses the quality of different areas of information presented and the quality of team behavior. Those variables are assessed using a standardized behavioral marker system, with descriptive end points at 1 (poor information quality/teamwork), 3 (average information quality/teamwork), and 5 (excellent information quality/teamwork) [16]. Psychometric studies showed adequate inter-rater reliability as well as concurrent validity [8, 18].

An initial sample of three MDTMs (assessed in September 2014) was used to pilot test and adapt the measure. Since the observers in our study were psychologists (SD, PH), we eliminated two variables that require medical judgments (“point in treatment”, “pathological information”), and the variables assessing the quality of contributions from different specialist groups. We also eliminated the item “meeting site”, since all MDTMs were held in the same room.

This led to an adapted version with six variables that assess the presented information on the case-level: 1) quality of case history, 2) quality of radiological information, 3) quality of information on comorbidities, 4) whether it was presented, whether the case was palliative, 5) quality of psychosocial information, and 6) quality of information on the patient’s views and preferences. Furthermore, three variables assess the quality of team processes on the case-level: 1) quality of MDTM chair behavior, 2) quality of team behavior, and 3) medical and treatment uncertainty during the case discussion. In this measure, high quality is generally operationalized as information being presented with a high level of comprehensiveness, elaborateness, and proximity to the patient (i.e. first-hand rather than second-hand knowledge). Medical correctness of the information and accordance with CPGs was not assessed. The lowest rating for items assessing the quality of the presented information was operationalized as no information being presented. With the exception of whether the discussed case was palliative or not palliative (dichotomous rating), the variables were rated on five-point Likert-scales (1 = no information/lowest quality to 5 = highest quality). Anchoring descriptions were elaborated for the scores 1, 3, and 5 for each variable (cp. Table 1) and discussed and refined throughout the adaptation process. The scores 2 and 4 were not explicitly elaborated and given, if the observer assessed the quality as between 1 and 3 or between 3 and 5 respectively. Additionally, the duration of discussion for each individual case and the number of active participants in the discussion of each individual case were assessed on the case-level. On the session level, the specialization and date of the MDTM, the duration of the session, the number of attending professionals, and the number of cases discussed in this session were noted.

**Table 1:** Description of the variables of the adapted measure

Variable	(Likert-)Scale	Description
Quality of case history	5	Fluent, comprehensive case history: Listing of name, age, major health problem, family diseases, medications
	3	Partial case history
	1	No case history
Quality of radiological information	5	Radiological images were shown and discussed during case discussion
	3	Radiological information from a report/account
	1	No provision of radiological information
Quality of information on comorbidities	5	Comprehensive first-hand knowledge of past medical history or performance status Listing of further diseases
	3	Vague first-hand knowledge or good second-hand knowledge of past medical history or performance status
	1	No information on past medical history or performance status
Palliative case (no/yes)	0	The case was not explicitly defined as palliative
	1	The case was explicitly defined as palliative
Quality of psychosocial information	5	First-hand knowledge and detailed consideration of information on patient’s personal and social circumstances: - profession - marital status, children - living arrangements First-hand knowledge and detailed consideration of patient’s psychological issues: - psychological problems - family problems - psychological disorders
	3	Vague first-hand knowledge or good second-hand knowledge of patients’ personal circumstances, social and psychological issues
	1	No information on patients’ personal circumstances, social and psychological issues
Quality of information on the patient‘s views	5	Comprehensive knowledge and detailed consideration of patient’s wishes or opinions regarding treatment: Someone who has met the patient presents their views/preferences/holistic needs
	3	Vague first-hand knowledge or good second-hand knowledge of patient’s wishes or opinions regarding treatment
	1	No information on patient’s wishes or opinions regarding treatment
Number of active participants	Number of active participants contributing to the discussion	
Quality of MDTM chair behavior	5	Good leadership enhanced team discussion and decision making: - Leader encouraged full participation of all team members - Showed assertive behavior - Demonstrated ability to resolve conflict - Monitored and coordinated contributions of team members
	3	Leadership neither enhanced nor impeded team discussion and decision making
	1	Poor/inadequate leadership impeded team discussion and decision making: - Interrupted team members or behaved in a disrespectful manner - Participated reluctantly - Avoided conflict - Leader could not be identified
Quality of team behavior	5	Good communication between team members: - Open and inclusive team discussion - Offering of constructive criticism - Climate of respect and equality, harmony within the group - Team engagement - Group cohesion (more than group of individuals)
	3	Communication between team members neither good nor poor
	1	Poor communication between team members: - Reluctant contributions of team members - Interruption of team members - Destructive team discussion - Hostile climate and disharmony within the group - Poor team engagement and group cohesion
Medical and treatment uncertainty during the case discussion	5	Team members showed medical and treatment uncertainty about best treatment decision
	3	Some medical and treatment uncertainty about decision was shown, but decision for one option seemed clear
	1	Team members seemed to have same opinion regarding treatment decision, no further treatment options mentioned
Recommendation reached?	Y	Clear recommendation about treatment(s) was offered
	D	Recommendation was deferred to next MDTM
	N	No recommendation or recommendation unclear
Number of recommendations	Number of treatment recommendations	
Free text	Additional observer comments	
Minutes per case	Minutes spent on discussing each case	

For statistical analyses, the outcome was classified in three distinct categories: 1) one treatment recommendation reached, 2) more than one treatment recommendation reached, or 3) no treatment recommendation reached (including treatment recommendation deferred). This outcome was chosen as a minimum standard of MDTM output analogously to the considerations described in the introduction [3, 4]. No conclusions about the clinical correctness of the content of the recommendations can be drawn within this study.

The full original version of the MDT-MODe can be found online on the webpage of the Center for Patient Safety and Service Quality of the Imperial College London [19], and the adapted version can be found in the additional files of this paper (cp. Additional file 1).

### Data collection

Prior to the data collection, the responsible physicians for each MDTM were contacted via email and informed about the study. It was known prior to the observations that the room in which the MDTMs take place would be darkened for the screening of the electronic medical records and equipped with approximately 50 seats. The observations were carried out by one assessor (SD), who was present at all MDTM sessions. Data collection was carried out as non-participant observation [20], with SD seated in the back, attracting as little attention as possible.

SD and PH studied training material provided by Lamb et al. to become familiar with the rating scale, and PH (who had experience in observing MDTMs from a previous study) trained SD in non-participant observation at MDTMs during the initial observations. In September and October 2014, one researcher (SD) attended 29 MDTMs. The first three of those MDTMs were observed by two researchers (SD and PH) in order to evaluate inter-rater reliability. Observations were recorded on the adapted MDT-MODe form. During the period of data collection SD and PH met regularly in order to safeguard the quality of the observational process. This included the reflection of the observation process and of challenges (e.g., interaction with physicians at the MDTM) that emerged during observations.

### Data analysis

Inter-rater reliability was assessed by computing intra-class correlation coefficients (ICC). For the assessment of inter-rater reliability, data from the three observed sessions during the adaptation phase as well as from the three sessions with two observers after the adaptation phase (i.e., six sessions in total) was used.

For the calculation of descriptive statistics and logistic regression analyses, data from 29 sessions (not including the observations during the measure adaptation phase) was used. Two-level mixed logistic regression models, that were fitted with a random intercept varying across sessions, were used to identify factors that were associated with whether a treatment recommendation was given or not, and whether one or more recommendations were given (both categorical dependent variables). For both outcomes, the full model included the same set of session-level and case-level variables. The following session-level variables were taken into account: 1) specialization of the MDTM, 2) duration of the session, 3) number of attending professionals, and 4) number of cases discussed in this session. On the case-level, included variables were 1) quality of case history, 2) quality of radiological information, 3) quality of information on comorbidities, 4) whether it was presented, whether the case was palliative, 5) quality of psychosocial information, 6) quality of information on the patient’s views and preferences, 7) quality of MDTM chair behavior, 8) quality of team behavior, 9) medical and treatment uncertainty during the MDTM discussion, 10) number of active participants in the discussion of each individual case, and 11) duration of discussion for each individual case. This led to 15 variables in the full model.

In addition to each full model, we also calculated a stepwise model with backward selection, removing one variable at each step (based on the highest *p*-value of the estimated fixed coefficients) until only variables with *p* < .10 remained. In order to account for the explorative character of the study, no adjustment for multiple testing was used and all findings with a type I error rate below.10 are reported. We approximated the global amount of variation in the outcome explained by the independent variables through calculating *R* ^*2*^ *= 1-(logL*_*1*_ */logL*_*0*_ *)*, where *logL*_*1*_ and *logL*_*0*_ are the values of the log-likelihood function from the model with and without predictors, respectively (*McFadden’s R* ^*2*^, mathematically equivalent to the relative reduction in deviance).

Analyses were performed with SPSS version 22 (SPSS Inc., Chicago, IL) and the lme4 package in R [21].

## Results

### Inter-rater reliability of the measure

Inter-rater reliability coefficients were calculated for a total of 39 cases from six MDTM sessions for all variables that were not adapted, and a total of 14 cases from three MDTM sessions for all adapted variables. At least moderate agreement between two observers (Cohen’s Kappa/*ICC* ≥ .5) was reached for all independent variables, except for quality of radiological information (*ICC* = −.1), quality of information on comorbidities (*ICC* = .2), and quality of information on the patient’s views and preferences (*ICC* = .4). However, if only the three later sessions (i.e., after the adaptation phase) were considered, ICCs rose to at least moderate agreement (*ICC* = 1.0 for quality of radiological information, *ICC* = .8 for quality of information on comorbidities, and *ICC* = .5 for quality of information on the patient’s views and preferences). This suggested adequate learning curves between the raters and led us to including all variables into subsequent analyses.

### Characteristics of observed MDTMs

Descriptive and regression analyses were performed for a total of 249 case discussions from a total of 29 MDTM sessions. Large variation was found for all variables describing MDTMs on a session-level. The sessions lasted between six and 85 min (mean = 48, standard deviation (*SD*) = 17.5, median = 45, interquartile range (*IQR*) = 19). Between six and 45 professionals attended the sessions (mean = 18, *SD* = 8.8, median = 15, *IQR* = 7), and between one and 17 cases were discussed in one session (mean = 11, *SD* = 3.9, median = 12, *IQR* = 4).

MDTMs made a treatment recommendation in the majority of cases (185 of 249 cases, 74.3%). Mostly, one treatment recommendation was given (160 of 249 cases, 64.3%). However, in one third of all cases, MDTMs did not reach a single recommendation (87 of 249 cases, 34.9%). More than one treatment recommendation was given in 25 of 249 cases (10.0%). No treatment recommendation was given in 62 of 249 cases (24.9%). In two cases (0.8%) data was missing.

As presented in Table 2, case history (mean = 4.9; *SD* = .5) and radiological information (mean = 4.5; *SD* = 1.3) were presented on a high level of quality at the observed MDTMs. In 234 (94.4%) and 201 (81.0%) of 248 valid cases case history and radiological information, respectively, were rated with 5, indicating information being presented with highest quality (cp. Additional file 2 for table with frequencies of case-level ratings). Psychosocial information (mean = 1.5; *SD* = 1.0) and patient views (mean = 1.4; *SD* = 1.0) were presented with the lowest quality (including not being mentioned at all). In 198 (79.8%) and 214 (86.3%) of 248 valid cases psychosocial information and patient views, respectively, were rated with 1, indicating no such information being presented. In 40 (16.3%) of 246 valid cases it was presented that the case at hand was palliative. On average, the quality of the MDTM chair behavior was rated as poor by the assessor (mean = 1.9; *SD* = 1.2) with 144 cases (58.3%) being rated with 1, indicating lowest quality. The quality of team behavior was considered generally positive (mean = 4.4; *SD* = .9; 142 cases (57.5%) rated with 5). Compare Table 1 for examples of positive and poor MDTM chair and team behavior. The mean observed medical and treatment uncertainty during the case discussions was on a mid-level with a large standard deviation (mean = 2.9; *SD* = 1.7).

**Table 2:** Descriptive statistics of case-level variables (*N* = 249 cases^a^)

	Mean (SD)	Range
Rating of information presented		
Quality of case history	4.9 (0.5)	1–5
Quality of radiological information	4.5 (1.3)	1–5
Quality of information on comorbidities	2.1 (1.4)	1–5
Quality of psychosocial information	1.5 (1.0)	1–5
Quality of information on patient view	1.4 (1.0)	1–5
Rating of quality of team processes		
Quality of MDTM chair behavior	1.9 (1.2)	1–5
Quality of team behavior	4.4 (0.9)	1–5
Medical and treatment uncertainty during case discussion	2.9 (1.7)	1–5
Additional variables		
Duration of case discussion (in minutes)	4.4 (2.6)	1–15
Number of participating physicians per case	4.5 (1.6)	1–11

*SD* standard deviation. Ratings on a Likert-scales from 1 = lowest quality to 5 = highest quality

^a^Due to missing values number of cases analyzed per variable ranged from 245 to 249 cases

### Factors associated with no recommendation

Table 3 illustrates the results of the regression analysis assessing which variables had a significant influence on whether no treatment recommendation was given: 1) whether a case was discussed at some of the specialized MDTMs (each compared to the gynecological MDTM), 2) duration of the session, 3) duration of the case discussion, 4) quality of case history, and 5) quality of radiological information. In the full as well as the stepwise model, it was found that it was more likely that no recommendation was given in the liver and biliary tract MDTM (odds ratio (*OR*) = 4.41 in the stepwise model). In MDTMs with other specializations (i.e. lymphoma and myeloma, non-entity specific surgical, thorax, uro-oncological), it was also more likely that a recommendation was given, but results were statistically significant only in the stepwise model (cp. Table 3). With every 10-min-increase of the duration of the session, it was 1.32 times more likely that no treatment recommendation was given. With every increasing minute of the duration of the case discussion, it was 1.13 times more likely that no treatment recommendation was given (statistically significant only in the stepwise model). Case history (*OR* = 0.30) and radiological information (*OR* = 0.68) of higher quality reduced the likelihood of giving no recommendation. The models explained a fifth to tenth of the variation in the outcome (*R* ^*2*^ = .195 for the full and.099 for the stepwise model).

**Table 3:** Results of the mixed logistic regression predicting for which cases no recommendation was given (*N* = 249 cases in 29 sessions)

Predictor	Full model			Stepwise model		
	OR	95% CI	*P*	OR	95% CI	*P*
Dermatological vs. gyn. MDTM	2.22	0.09 to 57.79	.629			
Gastrointestinal vs. gyn. MDTM	0.40	0.01 to 16.15	.625			
Head and neck cancer vs. gyn. MDTM	1.60	0.36 to 7.07	.534			
Liver and biliary tract vs. gyn. MDTM	**4.81**	1.10 to 20.98	.037**	**4.41**	1.38 to 14.11	.013**
Lymphoma and myeloma vs. gyn. MDTM	3.10	0.25 to 37.91	.373	**6.62**	0.81 to 53.85	.077*
Neuro-oncological vs. gyn. MDTM	1.99	0.20 to 20.10	.559			
Non-entity-specific oncological vs. gyn. MDTM	0.39	0.02 to 6.09	.497			
Non-entity-specific surgical vs. gyn. MDTM	2.64	0.39 to 17.86	.319	**3.48**	0.80 to 15.15	.096*
Thorax vs. gyn. MDTM	7.89	0.57 to 109.22.	.123	**9.48**	0.98 to 92.02	.052**
Uro-oncological vs. gyn. MDTM	3.13	0.23 to 43.50	.395	**9.40**	1.13 to 78.25	.038**
Number of attending professionals (1 person increase)	1.05	0.94 to 1.18	.385			
Duration of session (10 min increase)	**1.97**	1.09 to 3.58	.024**	**1.32**	0.98 to 1.76	.071*
Number of cases discussed in this session (1 case increase)	0.81	0.62 to 1.05	.111			
Quality of case history^a^	**0.36**	0.15 to 0.84	.019**	**0.30**	0.13 to 0.68	.004**
Quality of radiological information^a^	**0.74**	0.54 to 1.02	.063*	**0.68**	0.51 to 0.90	.008**
Quality of information on comorbidities^a^	0.85	0.66 to 1.11	.226			
Presentation of whether case was palliative (dichotomous variable)	1.06	0.36 to 3.13	.910			
Quality of psychosocial information^a^	1.08	0.76 to 1.53	.686			
Quality of information on the patient’s views and preferences^a^	0.91	0.64 to 1.30	.614			
Number of active participants in the discussion of each individual case (1 person increase)	0.94	0.73 to 1.21	.605			
Quality of chair behavior^a^	0.79	0.54 to 1.17	.237			
Quality of team behavior^a^	0.97	0.60 to 1.55	.884			
Medical and treatment uncertainty during case discussion^a^	1.10	0.85 to 1.42	.459			
Duration of case discussion (1 min increase)	1.13	0.94 to 1.27	.154	1.13	1.00 to 1.27	.067*

*OR* Odds ratio, *CI* Confidence interval, *gyn* gynecological

Bold typesetting of OR indicates statistical significance

*Indicates *p* < .10

**Indicates *p* < .05

^a^Indicates 1 step increase

### Factors associated with the number of recommendations

As illustrated in Table 4, medical and treatment uncertainty during the case discussion had a significant influence on whether multiple treatment options were recommended in the stepwise as well as the full model. The recommendation of multiple options was 2.16 times more likely, if medical and treatment uncertainty increased by one point on the Likert-scale (provided that all other factors are held constant). Additionally, if a case was discussed in the gastrointestinal (only in the stepwise model) or the neuro-oncological (only in the stepwise model) it was more likely that more than one recommendation was given compared to in the gynecological MDTM. The models explained around a third of the variation in the outcome (*R* ^*2*^ = .372 for the full and.308 for the stepwise model).

**Table 4:** Results of the mixed logistic regression predicting for which cases more than one option was recommended (*N* = 185 cases in 28 sessions)

Predictor	Full model			Stepwise model		
	OR	95% CI	*P*	OR	95% CI	*P*
Dermatological vs. gyn. MDTM	0.95	0.01 to 93.97	.981			
Gastrointestinal vs. gyn. MDTM	288.58	0.28 to >999.00	.109	**7.36**	1.38 to 39.35	.020**
Head and neck cancer vs. gyn. MDTM	1.94	0.17 to 22.07	.593			
Liver and biliary tract vs. gyn. MDTM	1.99	0.20 to 19.57	.552			
Lymphoma and myeloma vs. gyn. MDTM	4.85	0.17 to 135.52	.351			
Neuro-oncological vs. gyn. MDTM	2.81	0.12 to 68.75	.524	**5.39**	1.24 to 23.45	.025**
Non-entity-specific oncological vs. gyn. MDTM	5.45	0.08 to 386.63	.433			
Non-entity-specific surgical vs. gyn. MDTM	14.44	0.29 to 730.38	.181			
Thorax vs. gyn. MDTM	1.28	0.00 to >999.00	.944			
Uro-oncological vs. gyn. MDTM	21.27	0.31 to >999.00	.154			
Number of attending professionals (1 person increase)	0.90	0.71 to 1.13	.352			
Duration of session (10 min increase)	0.82	0.36 to 1.84	.630			
Number of cases discussed in this session (1 case increase)	1.20	0.82 to 1.76	.347			
Quality of case history^a^	0.64	0.02 to 26.76	.813			
Quality of radiological information^a^	0.74	0.39 to 1.41	.359			
Quality of information on comorbidities^a^	1.12	0.79 to 1.59	.532			
Presentation of whether case was palliative (dichotomous variable)	0.52	0.11 to 2.42	.399			
Quality of psychosocial information^a^	0.95	0.57 to 1.60	.836			
Quality of information on the patient’s views and preferences^a^	1.10	0.72 to 1.69	.661			
Number of active participants in the discussion of each individual case (1 person increase)	0.89	0.60 to 1.33	.571			
Quality of chair behavior^a^	1.24	0.68 to 2.26	.704			
Quality of team behavior^a^	1.32	0.58 to 3.01	.508			
Medical and treatment uncertainty during case discussion^a^	**2.12**	1.39 to 3.23	.001**	**2.16**	1.48 to 3.14	<.001**
Duration of case discussion (1 min increase)	1.13	0.94 to 1.43	.203			

*OR* Odds ratio, *CI* Confidence interval, *gyn* gynecological

Bold typesetting of OR indicates statistical significance

*Indicates *p* < .10

**Indicates *p* < .05

^a^Indicates 1 step increase

## Discussion

This study assessed the process quality of decision-making in MDTMs using a systematic observational assessment tool. Cancer-specific medical information was presented with the highest quality, while patient views and psychosocial information as well as information on comorbidities were presented with lower quality (often meaning that they were not presented at all). In the majority of cases, one treatment recommendation was given. The specialization of the MDTMs was shown to be associated with the recommendation outcome in several cases. Higher quality of case history and radiological information made it more likely that a recommendation was given. Time-related factors (i.e., duration of session and duration of case discussion) were also found to be interrelated with the outcomes. A higher level of medical and treatment uncertainty during the discussion was associated with a higher probability of giving more than one treatment recommendation.

Our results are consistent with other studies that also found that medical information was predominantly presented and/or presented with high quality at MDTMs, whereas psychosocial information and patient views were often not presented and/or presented with low quality [10, 11, 15]. We did not find MDTMs to be in line with the principles of patient centered care. This finding seems to persist despite health policy developments calling for a more patient-centered approach [22]. Additional studies are needed to explore how certification and quality management processes in hospitals affect the adherence to CPGs and, as a consequence, influence what is presented at MDTMs. The omission of psychosocial information and patient views may lead to physicians overlooking important additional attributes of a specific patient that may interfere with a planned treatment approach. This in turn can be an obstacle to a successful implementation of the treatment recommendation, as was found in previous studies [13].

In our data, one single treatment recommendation was given for the majority of cases discussed. It has been argued before that limited time and resources make patient-centered MDTM work hard to achieve [23]. Presenting only medical information might facilitate the agreement on a treatment recommendation in the majority of cases. This, as a consequence, might lead to more easily reaching one single recommendation for each case as well as shorter case discussions and shorter MDTM sessions. If additional factors such as psychosocial information or patient views would be taken into account, this might lead to physicians having more divergent opinions about the most appropriate treatments. Therefore, high workload and time pressure might be explanations for physicians being constrained to predominantly presenting medical information and reaching one single recommendation for most cases at MDTMs. Further studies are needed to further explore, why the patient perspective is often not presented and how MDTM recommendations are incorporated into subsequent clinical processes.

While the defined aim of MDTMs is to make treatment recommendations, those should not be made if information to thoroughly evaluate the case is lacking. Also, while one treatment recommendation might be the best way if there is a clear-cut best treatment recommendation, giving more than one recommendation at the MDTM might be helpful for patients as well as physicians if there is more than one suitable evidence-based treatment recommendation. This is especially important, since another study found considerable discrepancies between differently specialized physicians in their treatment recommendations for the same patient cases [24].

We found in this study that a higher level of medical and treatment uncertainty during the case discussions at the MDTMs increased the probability that more than one treatment recommendation was given. One could speculate that those might be the cases lacking a clear-cut CPG recommendation.

If one is aiming to implement a patient-centered approach and shared decision-making between physician and patient, it might be worthwhile to reflect critically the way MDTMs are currently executed. In line with the findings of the study at hand, we argued in another paper that “the current structure of MDTMs in Germany serves as a barrier to the implementation of SDM” [10]. As a substantial measure, the presence of patients or patient advocates at MDTMs could support adequate representation of patients’ views and relevant psychosocial information in MDTM discussions [25, 26]. Also, describing more than one treatment recommendation in case of medical uncertainty might give the treating physician and the patient more chance to weigh treatment options and find the best option in accordance with the patient’s preferences. This has also been argued for by other researchers [25]. A study in breast cancer found that almost half of the physicians viewed it as mandatory to implement MDTM recommendations in the subsequent consultation with the patient [27]. Documenting in the patient’s medical record if there was uncertainty during the MDTM discussion might be helpful for the treating physician to evaluate the recommendation. Furthermore, a change towards MDTM recommendations not being viewed as mandatory by treating physicians might give more room for subsequent discussion of treatments with the patient. Further investigations should assess how MDTM recommendations are brought back to the patients after the MDTM.

Different specializations of MDTMs were found to differ in how often they give no, one or more than one recommendations. One might speculate that cases might be more complex or CPGs might be more clear-cut for some specializations than for others. More research is needed to look into possible explanations for differences between MDTMs with different specializations.

A key strength of this study is that this was to our knowledge the first study that systematically examined decision-making processes at a large scale (*N* = 249 case discussions in 29 MDTMs). Moreover, the data was not collected for a single specialization of MDTMs, but for 11 different specializations of MDTMs, allowing the generalization of our findings to a large group of specializations of MDTMs. However, generalizability to other institutions and countries is a limitation of this study. Due to the fact that this was a single center study conducted in one comprehensive cancer center, further research is needed to discover whether our findings are applicable across cancer care institutions nationally and internationally. It is also important to keep in mind that the observations were carried out by psychologists, limiting the validity of assessments regarding specialist medical issues.

The number of cases for the evaluation of inter-rater reliability was quite low (*N* = 39 for not adapted variables, *N* = 14 for adapted variables), and the inter-rater reliability requirements for variables to be incorporated into subsequent analyses were set relatively loose. Due to appropriate learning curves in terms of inter-rater agreement, we believe that the inclusion of those variables was nevertheless fruitful. Regarding the interpretation of the mixed logistic regression, we might have overlooked some interrelations due to low statistical power and might have identified some spurious findings due to the liberal significance level at the same time. Thus, replication of the main findings is needed before firm conclusions can be drawn.

## Conclusion

This exploratory study including different specializations of MDTMs and the rigorous statistical analyses led to a set of interesting new results that enable a better understanding of decision-making processes at MDTMs. The quality of different aspects of information was observed to differ greatly (i.e. high quality cancer-specific medical information, low quality information on patient views and psychosocial information). Whether no, one or more than one recommendations were given varied substantially between different specializations of MDTMs. The quality of certain information (i.e. quality of case history and quality of radiological information) and time-related variables were also associated with the recommendation outcome. Medical and treatment uncertainty during discussions was related to giving more than one recommendation. Some of those aspects seem modifiable, which offers possibilities for the reorganization of MDTMs. MDTMs could include more in depth discussion of the patient perspective as well as of uncertainties. Also, time constraints will have to be tackled, if one wants to reorganize MDTMs into a forum that enables patient-centered decision-making.

## Additional files

Additional file 1: Adapted version of the MDT-MODe: Rating scale for the quality of decision-making processes in MDTMs. (PDF 185 kb)

Additional file 2: Frequencies of ratings for case-level variables. (*N* = 249 cases). (PDF 14.8 kb)

## Abbreviations

CI: Confidence Interval
CPG: Clinical Practice Guidelines
DFG: German Research Foundation (German: Deutsche Forschungsgemeinschaft)
gyn: gynecological
ICC: Intra-Class Correlation Coefficient
LK: Levente Kriston
MDT-MODe: Multidisciplinary Tumor Board Metric for the Observation of Decision-Making
MDTMs: Multidisciplinary Team Meetings
OR: Odds Ratio
PH: Pola Hahlweg
SD: Sarah Didi
SD: Standard Deviation
UCCH: University Cancer Center Hamburg
YN: Yvonne Nestoriuc
