# Supplementary material for: Process quality of decision-making in multidisciplinary cancer team meetings: a structured observational study
Source: BMC Cancer. 2017 Nov 17;17:772. doi: 10.1186/s12885-017-3768-5 (PMC5693525; doi:10.1186/s12885-017-3768-5)
Supplement: Supplementary file 1 — Adapted version of the MDT-MODe: Rating scale for the quality of decision-making processes in MDTMs. (PDF 185 kb) [file 12885_2017_3768_MOESM1_ESM.pdf]

Adapted version of the MDT-MODe: Rating scale for the quality of decision-making processes in MDTMs

Tumor board specialization:

Date:

Nr. of participants:

Place:

Start time:

End time:

	Information						Team processes				Outcome			
#	History	X-ray	Co-morbidity	Palliative case (0/1)	Psy/Soc	Patient view	Nr. of contributions	Chair	Team	Uncertainty	Y/D/N	Nr.of recommen-dations	Free text	Min/ case
1														
2														
3														
4														
5														
6														
7														
8														
9														
10														
11														
12														
13														
14														
15														
16														
17														
18														

**Quality of case history („history“)**	**5**	Fluent, comprehensive case history:<br>Listing of name, age, major health problem, family diseases, medications	**Quality of radiological information (“x-ray”)**	**5**	Radiological images were shown and discussed during case discussion
	**3**	Partial case history		**3**	Radiological information from a report/account
	**1**	No case history		**1**	No provision of radiological information
**Quality of information on comorbidities (“comorbidity”)**	**5**	Comprehensive first-hand knowledge of past medical history or performance status<br>Listing of further diseases	**Palliative case (0/1)**	**0**	The case was **not** explicitly defined as palliative
	**3**	Vague first-hand knowledge or good second-hand knowledge of past medical history or performance status		**1**	The case was explicitly defined as palliative
	**1**	No information on of past medical history or performance status			
**Quality of psychosocial information („Psy/Soc“)**	**5**	First-hand knowledge and detailed consideration of information on patient’s personal and social circumstances:<br>**- profession - marital status, children - living arrangements**<br><br>First-hand knowledge and detailed consideration of patient’s psychological issues :<br>**- psychological problems - family problems - psychological disorders**	**Quality of information on the patient‘s views (“patient view”)**	**5**	Comprehensive knowledge and detailed consideration of patient’s wishes or opinions regarding treatment:<br>Someone who has met the patient presents their views/preferences/holistic needs
	**3**	Vague first-hand knowledge or good second-hand knowledge of patients’ personal circumstances, social and psychological issues		**3**	Vague first-hand knowledge or good second-hand knowledge of patient’s wishes or opinions regarding treatment
	**1**	No information on patients’ personal circumstances, social and psychological issues		**1**	No information on patient’s wishes or opinions regarding treatment
**Quality of MDTM chair behavior („chair“)**	**5**	Good leadership enhanced team discussion and decision making:<br>**- Leader encouraged full participation of all team members**<br>**- Showed assertive behavior**<br>**- Demonstrated ability to resolve conflict**<br>**- Monitored and coordinated contributions of team members**	**Quality of team behavior (“team”)**	**5**	Good communication between team members:<br>**- Open and inclusive team discussion**<br>**- Offering of constructive criticism**<br>**- Climate of respect and equality, harmony within the group**<br>**- Team engagement**<br>**- Group cohesion (more than group of individuals)**
	**3**	Leadership neither enhanced nor impeded team discussion and decision making		**3**	Communication between team members neither good nor poor
	**1**	Poor/inadequate leadership impeded team discussion and decision making:<br>**- Interrupted team members or behaved in a disrespectful manner**<br>**- Participated reluctantly**<br>**- Avoided conflict**<br>**- Leader could not be identified**		**1**	Poor communication between team members:<br>**- Reluctant contributions of team members**<br>**- Interruption of team members**<br>**- Destructive team discussion**<br>**- Hostile climate and disharmony within the group**<br>**- Poor team engagement and group cohesion**
**Medical and treatment uncertainty during the case discussion (“uncertainty”)**	**5**	Team members showed medical and treatment uncertainty about best treatment decision	**Recommendation reached? (“Y/D/N”)**	**Y**	Clear recommendation about treatment(s) was offered
	**3**	Some medical and treatment uncertainty about decision was shown, but decision for one option seemed clear		**D**	Recommendation was deferred to next MDTM
	**1**	Team members seemed to have same opinion regarding treatment decision, no further treatment options mentioned		**N**	No recommendation or recommendation unclear
**Number of active participants (“Nr. of contributions”)**	Number of active participants contributing to the discussion		**Number of recommendations**	Number of treatment recommendations	
**Minutes per case (“Min/case”)**	Minutes spent on discussing each case		**Free text**	Additional observer comments	
