# Supplementary material for: Process quality of decision-making in multidisciplinary cancer team meetings: a structured observational study
Source: BMC Cancer. 2017 Nov 17;17:772. doi: 10.1186/s12885-017-3768-5 (PMC5693525; doi:10.1186/s12885-017-3768-5)
Supplement: Supplementary file 2 — Frequencies of ratings for case-level variables. (N = 249 cases). (PDF 14.8 kb) [file 12885_2017_3768_MOESM2_ESM.pdf]

Additional file B. Frequencies of ratings for case-level variables (N=249 cases[b]).

	Frequency (%) of ratings (on five-point Likert scale)				
	1 (lowest quality)	2	3	4	5 (highest quality)
Rating of information presented					
Quality of case history	2 (0.8%)	1 (0.4%)	7 (2.8%)	4 (1.6%)	234 (94.4%)
Quality of radiological information	27 (10.9%)	0 (0.0%)	7 (2.8%)	13 (5.2%)	201 (81.0%)
Quality of information on comorbidities	149 (60.1%)	0 (0.0%)	53 (21.4%)	21 (8.5%)	25 (10.1%)
Quality of psychosocial information	198 (79.8%)	2 (0.8%)	34 (13.7%)	10 (4.0%)	4 (1.6%)
Quality of information on patient view	214 (86.3%)	0 (0.0%)	11 (4.4%)	17 (6.9%)	6 (2.4%)
Rating of quality of team processes					
Quality of MDTM chair behavior	144 (58.3%)	19 (7.7%)	47 (19.0%)	32 (13.0%)	5 (2.0%)
Quality of team behavior	5 (2.0%)	9 (3.6%)	18 (7.3%)	73 (29.6%)	142 (57.5%)
Medical and treatment uncertainty during case discussion	93 (37.8%)	9 (3.7%)	57 (23.2%)	11 (4.5%)	76 (30.9%)

Note. [b] Due to missing values number of cases analyzed per variable ranged from 246 to 249 cases.
